# Phycobiliprotein Extract from *Arthrospira platensis* Boosts Immune Function in Pacific Oysters (*Magallana gigas*)

**DOI:** 10.3390/md23090355

**Published:** 2025-09-10

**Authors:** Aleksandra Andreyeva, Tatyana Kukhareva, Anastasiya Tkachuk, Maria Podolskaya, Elina Chelebieva, Andrey Borovkov

**Affiliations:** 1 Laboratory of Ecological Immunology of Aquatic Organisms, A.O. Kovalevsky Institute of Biology of the Southern Seas of RAS, 119991 Moscow, Russia; 2 Department of Biotechnology and Phytoresources, A.O. Kovalevsky Institute of Biology of the Southern Seas of RAS, 119991 Moscow, Russia

**Keywords:** the Pacific oyster, phycobiliproteins, immunomodulation, antioxidant activity, aquaculture

## Abstract

The utilization of functional feeds in oyster hatcheries to reduce disease-related issues and improve health in the prespawning period is expected to become essential in the near future. In the present study, an aqueous extract of phycobiliproteins (CBPs) sourced from the cyanobacterium *Arthrospira platensis* was tested as an immunomodulatory agent in the Pacific oyster (*Magallana gigas*). Adult oysters were given three distinct treatments with the aqueous extract of CBPs (2, 20 or 80 μg/mL) for 24–96 h. In vivo analysis demonstrated that the extract of CBPs enhanced phagocytosis, lysosomal content and mitochondrial membrane potential levels, but inhibited the production of reactive oxygen species in hemocytes of oysters. Higher concentrations of the extract (80 μg/mL) had a more rapid effect on phagocytosis, with significant differences found after the first 24 h of the experiment. Lower concentrations of the extract (2 μg/mL) enhanced the phagocytic activity of hemocytes at later stages of its administration. Additionally, the expression profiles of the *hsp70* and *hsp90* genes were monitored in gills from oysters exposed to the extract at concentrations of 2, 20 and 200 μg/mL for 48 h, considering their roles in regulating the innate immune system in bivalves. The results show that *hsp70* expression was down-regulated during the first 24 h of administration, whereas it recovered to control levels after 48 h. In contrast, the expression levels of *hsp90* were up-regulated throughout the entire period of extract administration. Combined, the results of the present study show that the aqueous extract of CBPs from *A. platensis* can rapidly enhance the cellular immune response in Pacific oysters, and could potentially be used as an immunomodulator in bivalve hatcheries.

## 1. Introduction

Shellfish farming is a rapidly growing food production sector that is essential for meeting the increasing demand for food due to the growing human population. One of the most valuable species in this sector is the Pacific oyster (*Magallana gigas*), which plays a significant role in global aquaculture [1]. However, due to environmental stressors and global climate change, oyster farming faces serious challenges related to survival in hatcheries and grow-out ponds, as well as vulnerability to diseases and poor production [2,3,4,5,6]. In response to these issues, intensive research is being conducted to reduce the mortality caused by diseases and changing ocean conditions [7]. As oysters undergo spawning during a limited period of the year in their natural environment, the development of a reliable source of spat can help to expand the bivalve aquaculture industry [8]. Hatcheries play a crucial role in the global oyster aquaculture industry. Before spawning, adult oyster broodstock are removed from their natural environment and kept indoors for up to 3 months indoors under controlled pH levels, temperature, salinity and photoperiod [9,10]. Before being moved to the hatchery, the oyster shells are cleaned and the mollusks are exposed to a low-salinity environment to remove epibionts and parasites [10]. These manipulations, along with changes in environmental conditions, can cause acute stress for the oysters which can affect their health and fitness [11,12,13,14,15,16,17]. Despite increasing demand from aquaculture farms, the mechanisms by which oyster broodstock adapt to a farmed environment are not well understood.

Improving nutrition in oyster aquaculture by incorporating health-promoting biologically active substances into their diet during the prespawning period holds promise for commercial applications in hatcheries [18,19]. Previous studies have shown that microalgae and their derivatives are a valuable source of various bioactive compounds that can enhance immunity in aquaculture [20]. Biomolecules derived from microalgae have been shown to have positive effects on growth performance, immunostimulation, and antioxidant and anti-inflammatory activities [21]. The nutritional value of microalgae has also been demonstrated in commercially important fish species. For example, rainbow trout (*Oncorhynchus mykiss*, Walbaum, 1792), gilthead seabream (*Sparus aurata* Linnaeus, 1758), Senegalese sole (*Solea senegalensis* Kaup, 1858) and tilapia (*Oreochromis niloticus*) experienced higher immunity and resistance to bacterial pathogens when some microalgae compounds (derived from *Chlorella* sp., *Tetraselmis* sp., *Navicula* sp., *Phaeodactylum tricornutum*, *Porphyridium cruentum*, *Nannochloropsis gaditana*, *Dunaliella salina*, *Lobosphaera* sp., *Schizochytrium* sp. and *A. platensis*) were added to fishmeal [22,23]. Similarly, growth results (body length, weight, growth rate) for guppy fish (*Poecilia reticulata*) fed with diets containing phycocyanin extracted from *Spirulina platensis* were significantly higher than those for the control group [24]. The application of microalgal biomolecules in the diets and feeds used in the invertebrate aquaculture industry has also been intensively explored. A feeding study on whiteleg shrimp (*Litopenaeus vannamei*) showed a significant increase in the non-specific immunity and a higher survival rate after infection with *Vibrio harveyi* in shrimps that received docosahexaenoic acid and arachidonic acid in their diet [25]. The resistance to bacterial infection with *A. hydrophila* in giant river prawns (*Macrobrachium rosenbergii*) was improved when they were fed a diet with 2–8% replacement of *Chlorella vulgaris* microalgae [26].

In bivalve aquaculture, the use of functional algal feed additives in hatchery diets is still limited, despite microalgae being a primary food source for these invertebrates. There are some issues with using microalgae as a replacement for regular diets in aquaculture, such as their poorly digestible cell walls and the risk of culture contamination. Therefore, the intake of their derivatives as biologically active compounds with health-promoting effects is preferred [27,28]. *Arthrospira plathensis* (spirulina) is commonly used as a source of several pigments, including zeaxanthin, carotene and phycobiliproteins, that are commonly added to diets as feed additives in aquaculture [29]. Phycobiliproteins (CBPs) are promising components of functional feeds, which are known to have anti-inflammatory, antioxidant and immune-enhancing effects in various commercially important species [30,31]. However, there is still a lack of fundamental knowledge on the effects of spirulina and its derivatives on the health of economically important bivalve species and, in particular, the Pacific oyster.

To better understand the potential use of CBPs as a functional feed additive in bivalve aquaculture—specifically, for *M. gigas*—we investigated the effects of an aqueous extract of CBPs from *A. plathensis* on oyster cellular immunity parameters and the expression levels of stress-related and regulatory immune genes at different concentrations.

## 2. Results

**Figure 1:**
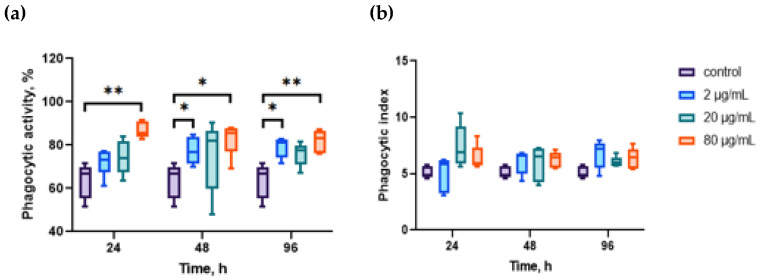
Effects of aqueous extract of CBPs derived from *A. plathensis* on phagocytosis of NR-conjugated zymosan particles. (**a**) Effects of different concentrations of the extract on phagocytic activity of hemocytes; (**b**) effects of the extract on phagocytic index of hemocytes. Data are the mean ± SE of 10 experiments in triplicate. *, *p* ≤ 0.05; **, *p* ≤ 0.01.

The effects of the aqueous extract of CBPs on hemocyte phagocytosis are reported in Figure 1. A significant increase in phagocytic activity (PA) was observed after exposure to 80 μg/mL for 24 h, which lasted throughout the entire duration of the experiment. Lower concentrations of the extract (2 μg/mL) also stimulated phagocytosis in hemocytes following incubation for 48–96 h (*p* ≤ 0.05, *n* = 10) (Figure 1a). Although the general phagocytic capacity of the hemolymph increased, the extract did not influence the individual phagocytic ability of hemocytes since we did not observe significant changes in the phagocytic index (PI) (Figure 1b).

**Figure 2:**
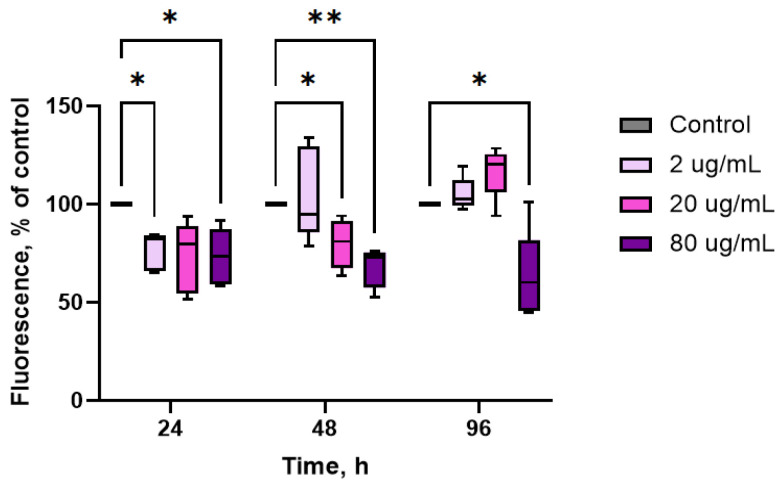
Effects of aqueous extract of CBPs derived from *A. plathensis* on ROS production by oyster hemocytes. Intracellular ROS production evaluated as fluorescence of DCF-DA, as described in the Methods. Data are the mean ± SE of 10 experiments in triplicate. *, *p* ≤ 0.05; **, *p* ≤ 0.01.

As shown in Figure 2, all concentrations of the extract induced a rapid decrease in intracellular ROS production, reaching a maximal increase of almost 2-fold with respect to controls at 80 μg/mL throughout the entire experiment (24–96 h) (*p* ≤ 0.05, *n* = 10). A significant decrease was also observed with lower extract concentrations (2–20 μg/mL): −25% after 24 h exposure to 2 μg/mL and −20% after 48 h exposure to 20 μg/mL (*p* ≤ 0.05, *n* = 10).

**Figure 3:**
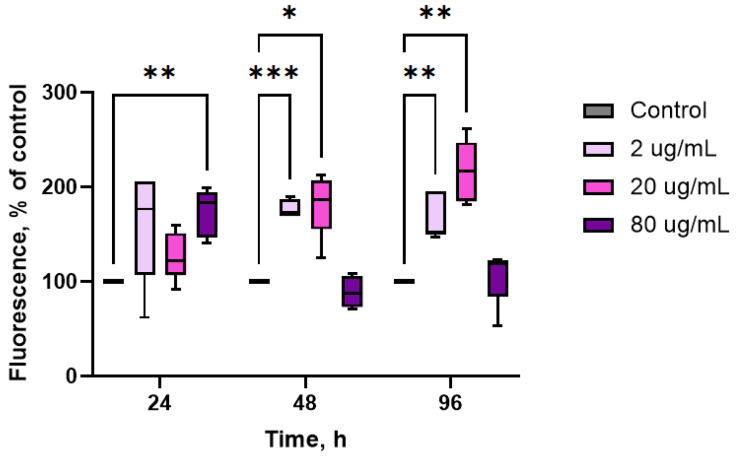
Effects of aqueous extract of CBPs derived from *A. plathensis* on the mitochondrial membrane potential of oyster hemocytes. Data are the mean ± SE of 10 experiments in triplicate. *, *p* ≤ 0.05; **, *p* ≤ 0.01; ***, *p* ≤ 0.001.

Changes in mitochondrial membrane potential (MMP) in hemocytes were evaluated via the fluorescence of R123 (Figure 3). The levels of R123 in control hemocytes were set as the standard MMP level. Administration of the extract resulted in a significant increase in hemocyte MMP levels, although there was a large variability in the time course of the extract-induced changes among the different concentrations used. The maximal increase in MMP was observed after 48–96 h (+82% and +115% respectively, *p* ≤ 0.05, *n* = 10). Additionally, the lowest concentrations of the extract enhanced mitochondrial respiration in hemocytes after 24–48 h of exposure. High concentrations of the extract (80 μg/mL) led to an increase in MMP levels after 24 h of exposure (*p* ≤ 0.05, *n* = 10); however, further administration of the extract did not affect this parameter.

**Figure 4:**
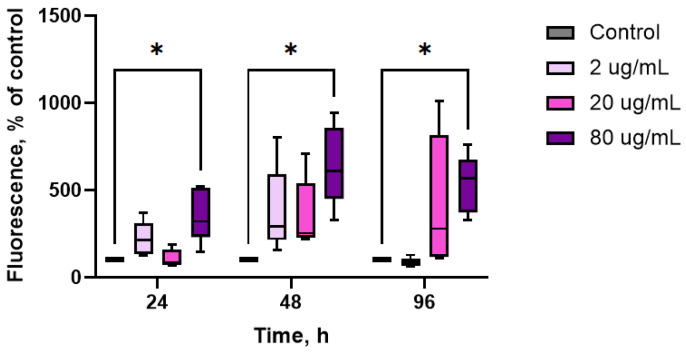
Effects of aqueous extract of CBPs derived from *A. plathensis* on the lysosomal content in oyster hemocytes. Data are the mean ± SE of 10 experiments in triplicate. *, *p* ≤ 0.05.

After incubating oysters with aqueous extract of CBPs at concentrations ranging from 2 to 20 μg/mL, there were no significant changes in the levels of lysosomal content in hemocytes throughout the entire period of the experiment (Figure 4). However, when the concentration was increased to 80 μg/mL, the lysosomal content was significantly elevated compared to the control group (*p* < 0.05, *n* = 10).

**Figure 5:**
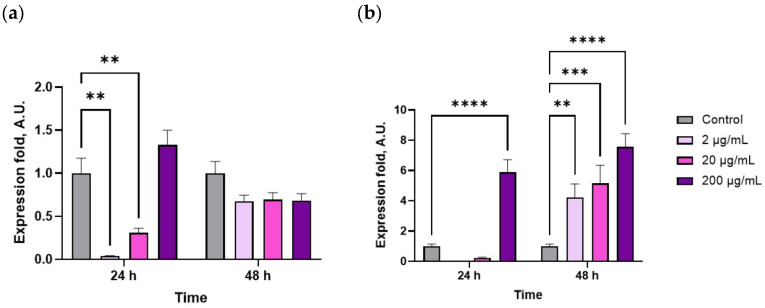
Relative mRNA expression levels of heat shock protein genes in the gills of the Pacific oyster *M. gigas* fed with graded levels of aqueous extract of CBPs for 24 h and 48 h: (**a**) Expression of *HSP70*; (**b**) expression of *HSP90* (two-way ANOVA followed by Dunnet’s multiple range test). **, *p* ≤ 0.01; ***, *p* ≤ 0.001; ****, *p* ≤ 0.0001.

The extract concentration and exposure duration significantly influenced the expression levels of *HSP70* and *HSP90* (*p* < 0.05, *n* = 10). In the gill tissue, exposure to 2–20 µg/mL of the extract for 24 h reduced *HSP70* expression (Figure 5a), with no significant change observed after 48 h. In contrast, *HSP90* expression increased markedly after 24 h, particularly at 200 µg/mL (Figure 5b) and, by 48 h, the levels were elevated 4–5-fold compared to control (*p* < 0.05, *n* = 10), indicating a sustained stress response.

## 3. Discussion

Previous studies have reported on the essential roles of spirulina and its pigments in enhancing the antioxidant and immune capacities of organisms. Dietary supplementation with spirulina-derived compounds has been shown to significantly improve growth performance, innate immunity, and disease resistance in various aquaculture species, including teleosts and crustaceans [32]. However, in bivalve hatcheries, feed formulations are still suboptimal and do not adequately support immunity or improve the health and productivity of spat and broodstock, despite being a significant cost item in production. The results of this study demonstrate that the aqueous extract of CBPs can rapidly enhance immune parameters in oysters when administered in vivo. Specifically, phagocytosis and lysosomal activity were increased in oyster hemocytes following administration of the CBP extract, and these effects were accompanied by an increase in the MMP of hemolymph cells. Additionally, a decrease in intracellular ROS formation levels in hemocytes was observed, indicating the antioxidant properties of the extract. Furthermore, we found that the aqueous extract of CBPs significantly influenced the expression of heat shock proteins in gills; specifically, *HSP70* expression was down-regulated after the first 24 h of the experiment, while *HSP90* expression was up-regulated throughout the duration of the experiment. Thus, the present study is the first to provide evidence on the immunomodulatory and antioxidant effects of CBPs in bivalve species.

In aquatic invertebrates including crustaceans and bivalves, the primary immune response is carried out by hemocytes, which phagocytose infectious agents in phagosomes and use a variety of intracellular killing mechanisms, such as an oxidative burst through excessive ROS formation and the synthesis of degradative enzymes like lysozymes [33,34]. The aqueous extract of CBPs significantly stimulated the phagocytic process, with the highest concentration (80 μg/mL) having the most rapid effect on the phagocytic ability of oyster hemocytes. However, lower concentrations (2 μg/mL) were more effective at later stages. As a stimulator of the immune system, spirulina has been shown to increase the macrophage phagocytic activity and activate T and B lymphocytes in mammals, as well as stimulating the production of humoral immune factors [35,36,37]. Considering aquatic invertebrates, the results of this study are consistent with the work of Lee et al. in 2021, who showed a dose-dependent increase in the PA of hemocytes in the whiteleg shrimp *L. vannamei* treated with phycoerythrin from *Colaconema* sp. [38]. A statistically significant increase in the PA of hemocytes was also observed in prawns (*Penaeus merguiensis*) fed with Spirulina powder [39]. Similarly, inclusion of C-phycocyanin in the diet of the Pacific white shrimp *P. vannamei* has been shown to enhance the expression of genes involved in the activation of the phenoloxidase pathway which, in turn, regulates non-specific immune responses including phagocytosis [30].

Along with increased phagocytosis, an increase in lysosomal activity was also observed, as indicated by the standard measurement of LysoTracker fluorescence in oyster hemocytes [40]. These results are consistent with previous observations of an increase in lysozyme activity in hemocytes of whiteleg shrimp (*L. vannamei*) fed spirulina-containing diets for a period of four weeks [41]. Additionally, similar increases in plasma lysozyme levels have been observed following dietary supplementation with *S. platensis* nanoparticles in Nile tilapia (*O. niloticus*) [42,43].

Non-stimulated hemocytes have been shown to produce higher levels of ROS in the cytoplasm, when compared to other types of somatic cells [44]. Although ROS produced by hemocytes are intended to destroy pathogens, their excessive formation can affect host immune cells under environmental stress [45,46]. In the present study, ROS production by oyster hemocytes was significantly reduced after addition of the aqueous extract of CBPs to the oyster’s diet (see Figure 2), indicating the antioxidant properties of this extract and its protective effect on the host’s immune system. The antioxidant activity of the CBPs was similar to that observed after exposure of hemocytes from the whiteleg shrimp *L. vannamei* to phycoerythrin from *Colaconema* sp. in vitro [38]. Similarly, the intake of dietary C-phycocyanin by the Pacific white shrimp *P. vannamei* resulted in a decrease in SOD mRNA levels in the gills, indicating a reduction in ROS production in tissues as reported by the authors [30]. Interestingly, the levels of lysosomal activity were significantly increased in hemocytes of oysters exposed to the aqueous CBP extract, suggesting that this dietary supplement may enhance certain immune responses in cells.

In invertebrates, including bivalves and insects, the immune defense has been shown to be energetically costly. This requires metabolic adaptation and increased energy consumption during the induced immune response [47,48]. Hemocytes, being the only circulating cells in the hemolymph, play a major role in inducing the immune response in the organism. Therefore, the functional state of hemocytes is thought to greatly influence the overall capacity of the host’s immune system. Our results show that administration of the aqueous CBP extract increased MMP levels in hemocytes. Mitochondria are directly involved in aerobic energy production, so we can assume that the extract increases the metabolic rate of oyster hemocytes. Considering that MMP levels decrease significantly during an immune response [49], we can conclude that the stimulating effect of the extract may be attributed to the enhanced metabolic capacity of hemocytes involved in the immune response.

The improved cellular immune parameters and hemocyte metabolism in *M. gigas* supplemented with CBPs might be linked to its stimulatory effect on immune regulatory signaling pathways. Administration of C-phycocyanin has been shown to significantly up-regulate the expression of prophenoloxidase (ProPO) and *HSP70* genes in gills of the Pacific white shrimp *P. vannamei* [30]; livers of the Nile tilapia *O. niloticus* [42] and *O. bidens* [50]; and hemolymph of the whiteleg shrimp *L. vannamei* [38]. These results are in line with our findings, as both *HSP70* and *HSP90* expression levels in the gills were influenced by administration of the aqueous extract of CBPs (see Figure 5). However, the potential mechanisms behind the inhibition or down-regulation, as well as the induction or up-regulation of HSPs in bivalves remain unclear. General mechanisms leading to reduced *HSP70* levels in the gills include down-regulation of protein synthesis, pathological effects and/or reduced energy (ATP) availability. However, we lack direct information about these processes [51]. Additionally, this response was only observed during the first 24 h of extract administration. In contrast, *HSP90* expression was up-regulated throughout the entire duration of the experiment. The induction of HSPs is considered a vital protective response that is conserved across organisms, which plays a crucial role in mounting innate immune responses against bacterial and viral infections [52]. Thus, the results of the present study likely suggest that the increase in *HSP90* induced by the aqueous extract of CBPs indicates its influence on molecular pathways regulating immune responses in oysters.

## 4. Materials and Methods

### 4.1. Test Substance

An aqueous extract of phycobiliproteins (CBPs) was obtained from *Arthrospira platensis* biomass. Spirulina strain IBSS-31 was provided by the Scientific and Educational Center for Collective Use “Collection of aquatic organisms of the World Ocean” of A.O. Kovalevsky Institute of Biology of the Southern Seas of RAS. The spirulina culture was grown in Zarrouk’s medium in open tanks [53,54].

The cultivation of *A. platensis* was conducted in a greenhouse-based algobiotechnological facility in Sevastopol, located at N. 44.61564° and E. 33.50463°, during the summer months. The photobioreactors consisted of 5 × 2.5-m square ponds lined with 200-micron polyethylene film, placed on a flat surface. The microalgae culture was grown in batch mode under natural lighting conditions. Illumination on the plant surface was monitored using a Li-250A light meter (LI-COR Biosciences, Lincoln, NE, USA) equipped with a Li-190R quantum sensor. The average total irradiation on the operating surface of the ponds during daylight hours was approximately 7.7 MJ/m^2^/day. During the growing period, temperatures ranged from 25 °C to 35 °C. To maintain consistent growth, the biomass was continuously mixed using electric aquarium pumps. The depth of the culture in the ponds was kept at 10 cm by adding fresh water daily until the initial level was restored. Throughout the trials, the pH level of the medium remained between 8 and 9.

To enhance pigment extraction, distilled water was added to the biomass, followed by two freeze–thaw cycles. Further extraction was carried out in cold distilled water (5 °C) for 24 h. The extract was separated from the biomass via centrifugation (Eppendorf 5430R, Leipzig, Germany, 6000 rpm, 10 min) and stored at −18 °C in the dark.

The pigment concentration in the extract was determined spectrophotometrically (UV-2600i spectrophotometer, Shimadzu Corporation, Kyoto, Japan) within the wavelength range of 400–800 nm. Optical density was measured at the absorption maxima of R-phycocyanin (615 nm), C-phycocyanin (620 nm) and allophycocyanin (650 nm), as well as at 750 nm to account for nonspecific absorption. The pigment concentration was calculated using the following equation [55]:

C − CBP = 0.166 × D620 − 0.091 × D650(1)

where D represents the optical density values at the respective wavelengths.

The CBP concentration in the extract ranged from 4000 to 4500 μg/mL.

### 4.2. Experimental Animals

Mature specimens of the Pacific oyster *M. gigas* (5 years old; mean body weight 72.1 ± 6.2 g; shell length 10.8 ± 1.9 cm; *n* = 350) were obtained from a commercial aquaculture farm (Sevastopol, 44.616014, 33.502248). The organisms were maintained in 50–70 L plastic tanks under controlled environmental conditions: water temperature 14–16 °C, pH 8.2, dissolved oxygen concentration 7–8 mg/L, salinity 17–18 psu and a 12 h photoperiod. Prior to experimental procedures, a 7-day acclimation period was implemented. Nutrition was provided daily using a monoculture of the microalga *Tetraselmis viridis* (Rouchijajnen, R.E. Norris, Hori, and Chihara, 1980), strain IBSS-25, supplied by the Department of Biotechnology and Phytoresources at the Federal Research Center IBSS. The feeding regimen consisted of a standardized daily ration of 10^9^ algal cells per individual. Throughout both acclimatization and experimental phases, a systematic water exchange protocol was maintained, wherein two-thirds of the tank volume was replaced daily to ensure efficient elimination of metabolic byproducts.

### 4.3. Feeding Experiment

After acclimation to laboratory conditions, the oysters were randomly divided in 4 groups (*n* = 350): control (no extract) and 3 experimental groups with the aqueous extract of CBPs added into tanks reaching final concentrations of 2 μg/mL, 20 μg/mL or 80 μg/mL. The range of concentrations used in the experiment was based on previous studies that did not reveal acute toxicity of the extract [56]. Oysters were exposed to the extract for 24, 48 and 96 h and their hemolymph was sampled for evaluation of cellular immune parameters.

For analysis of heat shock protein expression patterns, we used a modified experimental protocol. Oysters were exposed to the following extract concentrations: 2 μg/mL, 20 μg/mL or 80 μg/mL for 24–48 h. The higher concentration of 200 μg/mL was selected for gill tissue instead of 80 μg/mL due to the structural and functional adaptations of gills, including their barrier function and continuous environmental exposure, which confer greater stress tolerance compared to hemolymph cells, thereby enabling detection of maximal HSP induction under experimental feeding [57,58]. The 24–48 h exposure period was chosen because, although gills exhibit slower stress responses than isolated cells, peak HSP expression typically occurs within this timeframe, whereas prolonged exposure could induce nonspecific secondary effects that might obscure heat shock-specific responses [59].

### 4.4. Investigated Parameters

Hemocyte suspensions were prepared according to the methodology described by Andreyeva et al. (2021) [60]. Briefly, hemolymph samples (1.0–2.0 mL) were aseptically collected from the cardiac sinus of each oyster using sterile syringes. To ensure sufficient cellular material for analysis, hemolymph from two individuals was pooled, resulting in a total of 10 composite samples per group (*n* = 10). The samples were immediately processed by centrifugation at 500× *g* for 5 min at 10 °C (Eppendorf 5430R, Leipzig, Germany) to separate cells from plasma. The obtained cell pellets were resuspended in sterile filtered (0.2 μm) seawater to achieve a hemocyte concentration of 2–4 × 10^7^ cells/mL. Given the adhesive characteristics of bivalve hemocytes [61], all samples were maintained at 4 °C throughout the preparation and storage periods prior to analysis.

#### 4.4.1. Flow Cytometric Analysis

Hemocyte immune properties were assessed through flow cytometric analysis using a MACSQuant^®^ flow cytometer (Miltenyi Biotec, Bergisch Gladbach, Germany) equipped with a 488 nm excitation laser. Cellular parameters were acquired following standardized protocols, with a minimum of 10,000 events recorded per sample. All measurements were repeated three times. Data acquisition and preliminary analysis were conducted using the instrument’s software package (MACSQuantify™, version 2.13, Bergisch Gladbach, Germany), with subsequent analytical processing performed in triplicate to verify the measurements’ reproducibility. The gating strategy employed forward and side scatter parameters to discriminate distinct hemocyte populations, while fluorescence channels were utilized for functional characterization.

The quantification of reactive oxygen species (ROS) generation in hemocytes was performed through flow cytometric analysis of 2′,7′-dichlorodihydrofluorescein diacetate (DCF-DA) fluorescence. This non-fluorescent, cell-permeable compound serves as a sensitive indicator of intracellular ROS levels [62]. Following cellular uptake, endogenous esterases hydrolyze DCF-DA to its active form, 2′,7′-dichlorodihydrofluorescein (DCFH), which undergoes oxidation in the presence of ROS to yield the fluorescent product 2′,7′-dichlorofluorescein (excitation/emission maxima: 485/530 nm). Cells were incubated with 10 mM DCF-DA for 40 min prior to analysis. The intensity of fluorescence emission, measured as mean fluorescence intensity across the entire hemocyte population, was quantified in arbitrary units (a.u.).

The detection and quantification of lysosomal content in hemocytes were performed using LysoTracker Red (Lumiprobe, Moscow, Russia), a fluorescent membrane-permeable dye that selectively accumulates in lysosomal compartments. Hemocyte suspensions were incubated with LysoTracker Red at a final concentration of 1 µM. The mixtures were incubated for 30 min in darkness at room temperature (20 °C). The relative intracellular lysosomal content was quantified based on the red fluorescence intensity, measured using the FL3 detector of the flow cytometer and expressed in arbitrary units (a.u.).

Changes in the mitochondrial membrane potential of hemocytes were determined using the fluorescent stain Rhodamine 123 (Rh123) (Sigma Aldrich, Darmstadt, Germany). In particular, 1 mL of cell suspension was incubated with 10 μL Rh123. The final concentration of Rh123 in the probe was 0.1 mg mL^−1^. The fluorescence of cells was recorded in the FL 1 channel (green fluorescence) and expressed in arbitrary units (a.u.).

#### 4.4.2. Microscopic Observations

The phagocytic activity of hemocytes was assessed using fluorescence microscopy. Hemocyte suspensions were incubated with zymosan A pre-labeled with Nile red (NR) for 30 min in the dark at room temperature. The hemocyte:zymosan ratio was set as 1:20 (20 °C, 60 min, dark conditions) to facilitate particle internalization. Following incubation, cells were pelleted via centrifugation (500× *g*, 5 min, Eppendorf 5430R, Leipzig, Germany) and subjected to three consecutive washes with sterile seawater to eliminate non-internalized particles.

Microscopic analysis was performed using an Olympus CX43 fluorescence microscope (Olympus Corporation, Tokyo, Japan). The quantitative assessment included determination of two key parameters: (1) phagocytic activity (PA), expressed as the percentage of zymosan-positive cells among 1000 examined hemocytes per experimental group; and (2) phagocytic index (PI), calculated as the mean number of internalized zymosan particles per cell based on the analysis of 700 hemocytes per treatment group.

### 4.5. RNA Extraction and qRT-PCR Analysis of Gene Expression

For analysis of heat shock protein expression patterns, we used a modified experimental protocol. Oysters were exposed to the following extract concentrations: 2 μg/mL, 20 μg/mL or 80 μg/mL for 24–48 h. The higher concentration of 200 μg/mL was selected for gill tissue instead of 80 μg/mL due to the structural and functional adaptations of gills, including their barrier function and continuous environmental exposure, which confer greater stress tolerance compared to hemolymph cells, thereby enabling detection of maximal HSP induction under experimental feeding. The 24–48 h exposure period was chosen because, although gills exhibit slower stress responses than isolated cells, peak HSP expression typically occurs within this timeframe, whereas prolonged exposure could induce nonspecific secondary effects that might obscure heat shock-specific responses. Total RNA was isolated from *M. gigas* gill tissue using RNA Extran (Syntol, Moscow, Russia) to evaluate the expression profiles of the *HSP70* and *HSP90* genes. Following experimental exposure, gill samples (*n* = 10 per treatment group) were homogenized, and RNA was treated with DNase I to eliminate genomic DNA contamination. Purified RNA was eluted in 25 μL of nuclease-free water (Evrogen, Moscow, Russia) and quantified using a Qubit 4.0 fluorometer (Thermo Fisher Scientific, Waltham, MA USA). RNA integrity was verified via electrophoresis on a 1.5% agarose gel stained with ethidium bromide. First-strand cDNA synthesis was performed using the Mint-2 reverse transcription kit (Evrogen, Moscow, Russia) in a 15 μL reaction volume containing 1 μg of total RNA, following the manufacturer’s protocol. Gene-specific primers (Table 1; [63,64]) were used for quantitative real-time PCR (qRT-PCR) to measure relative transcript levels.

qRT-PCR was performed using a LightCycler 96 system (Roche, Grenzach-Wyhlen, Germany) with the qPCRmix-HS SYBR Green I kit (Evrogen, Moscow, Russia). Each 25 µL reaction contained 1 µL of cDNA, 0.5 µM of each primer and nuclease-free Milli-Q water. The thermal cycling protocol consisted of initial denaturation (95 °C, 30 s), followed by 40 cycles of denaturation (95 °C, 5 s), annealing (55 °C, 20 s) and extension (72 °C, 10 s), with a final extension step (72 °C, 7 min). Melting curve analysis was performed from 55 °C to 95 °C (0.5 °C/s increment) to confirm amplification specificity. All reactions were run in triplicate, including a no-template control (NTC) for each primer set. The amplification efficiency was determined using a cDNA serial dilution standard curve. Cycle threshold (Ct) values were automatically calculated, using the LightCycler software (v1.1, Roche, Grenzach-Wyhlen, Germany), at the point where fluorescence exceeded the baseline threshold. Gene expression levels were normalized to the reference gene Elongation factor 1 alpha (*EF1α*) of *M. gigas*, which exhibited stable expression across experimental conditions. Relative quantification was performed using the 2−ΔΔCT method [65].

### 4.6. Statistics

The statistical analyses and data visualization were conducted using GraphPrizm version 9 (GraphPad Software, San Diego, CA USA). No outliers were excluded from the datasets upon analysis and the construction of graphs. The Kolmogorov–Smirnov and Shapiro–Wilk tests were employed to ascertain the normality of the data, and the Levene test was performed to assess homogeneity. Any data that did not follow a normal distribution were normalized using the quantile normalization method. A two-way ANOVA with Dunnet’s HSD was conducted to evaluate the effects of time and extract concentration. The resulting *p*-values < 0.05, indicating that the observed differences were statistically significant. The data are reported as Mean ± SE.

## 5. Conclusions

The results of this study confirm that dietary supplements based on CBPs derived from spirulina can be successfully used as immunostimulants for bivalves in hatcheries. This study revealed that the aqueous CBP extract derived from *A. plathensis* has immune-regulating potential, as evidenced by the increases in cellular immune parameters and mitochondrial membrane potential in oyster hemocytes. Dietary supplementation with the aqueous CBP extract at a concentration of 80 μg/mL significantly enhanced cellular immune parameters, including phagocytic ability and lysosomal content of *M. gigas* hemocytes, following 24–96 h exposure periods. Lower concentrations of the extract (2–20 μg/mL) may require longer dietary supplementation to produce health-promoting effects in oyster broodstock. Additionally, dietary supplementation with CBPs improved the antioxidant capacity of *M. gigas* by reducing ROS formation by hemocytes. It was demonstrated that exposing oysters to the aqueous extract of CBPs significantly increased the expression of the *HSP90* gene and reduced the expression of *HSP70* in oyster gills. This increase is likely due to strengthening of the innate immune defense mechanisms, increasing the resilience of oysters to environmental stresses during broodstock conditioning in hatcheries. The results of this study are expected to help in developing functional feeds for oysters that can prevent disease and improve their health under hatchery conditions. While these findings are promising for hatchery-scale applications, future work must focus on optimizing the extraction process. Exploring strain selection and standardization will be crucial next steps to develop a commercially viable immunostimulant product for the oyster aquaculture sector.

## Figures and Tables

**Table 1:** Real-time quantitative PCR primers for heat shock protein genes and Elongation factor 1 alpha (*EF1α*) gene of the Pacific oyster *M. gigas*.

Gene Name	Nucleotide Sequence (5′–3′)		Efficiency (%)	Genbank Accession Number
Elongation factor 1 alpha (*EF1α*) (reference)	F	AGTCACCAAGGCTGCACAGAAAG	99.8	AB122066.1
	R	TCCGACGTATTTCTTTGCGATGT		
*HSP70*	F	AACGGTATCCTGAATGTGTC	101.1	AF144646
	R	CTTCTCGTCTTCCTGCTTG		
*HSP90*	F	CGAGGAAGCAGAAGCAGAG	98.8	AF144646
	R	ATGTCACCAGACGGTTAGATAC		

## Data Availability

Data will be made available on request.
